# Stepwise Excited-state Double Proton Transfer and Fluorescence Decay Analysis

**DOI:** 10.1007/s10895-022-03042-w

**Published:** 2022-10-22

**Authors:** Tomasz Wróblewski, Dzmitryi Ushakou

**Affiliations:** Institute of Exact and Technical Sciences, Pomeranian University in Słupsk, str. Arciszewskiego 22b, Słupsk, 76-200 Poland

**Keywords:** ESIPT, Stepwise ESIDPT, Fluorescence, Emission, Fluorescence decay kinetics

## Abstract

This work considers excited state intramolecular proton transfers (ESIPT) occurred in multiple hydroxyl-containing compounds with one proton transfer site in the normal form. If several hydroxyl groups are located close to each other in a molecule, then the ESIPT process can lead to the next one. A proton donor site in the first ESIPT will be a proton acceptor during the second reaction. Therefore, a number of consecutive excited state proton transfers can occur. This work deals with the case of two successive proton transfers occurred in the molecular system. Such process is called as a stepwise excited state intramolecular double proton transfer (stepwise ESIDPT). It leads to the formation of two molecular tautomers. Therefore, fluorescence of such compounds can contain different emission bands correspond to emission of normal form and two tautomers. In this work, a rigorous analysis of fluorescence decay kinetics has been made using the model with three species, including a normal molecular form and two tautomers. The work presents theoretical framework of fluorescence decay analysis of ESIDPT process taking into account three species emission. Theoretically, the stepwise proton transfers can be consisted of more than two ESIPT reactions. It depends on molecular structure and number of involved hydroxyl groups. Here, a formal analysis of fluorescence decay kinetics has been made in the case of a stepwise process consisting of two proton transfers. Moreover, the quantum-chemical calculations have been performed in the case of scutellarein. It is a multiple hydroxyl-containing flavone and, therefore, it can be applied as a model molecule to study stepwise intramolecular proton transfers. The hypothetical scheme of ESIDPT has been proposed for this compound.

**Graphical abstract:**

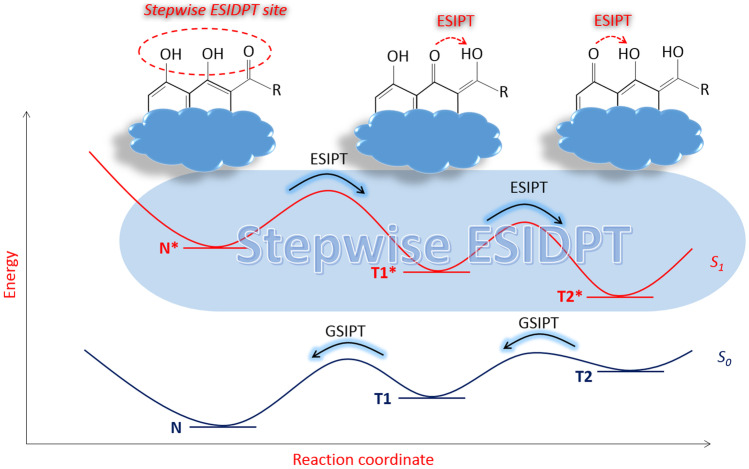

**Supplementary Information:**

The online version contains supplementary material available at 10.1007/s10895-022-03042-w.

## Introduction

Intramolecular proton transfer processes can lead to significant changes in photophysical properties of compounds. For example, the occurrence of excited state intramolecular proton transfer (ESIPT) can cause dual fluorescence emission due to presence of two different forms of molecules – normal and tautomer [1–4]. One of the first observations of an extremely large Stokes shift caused by excited state intramolecular proton transfer was made for salicylic acid in 1955 by Weller [5]. A year later, Weller reported the dual fluorescence caused by ESIPT experimentally observed in methyl salicylate [6]. Nowadays, due to their properties, compounds with ESIPT are used in a wide spectrum of applications, including production of luminescent materials [7], fluorescent chemosensors [8, 9], optoelectronic devices [10] and in particular solid state emitters [11], organic lasers [12] and white light-emitting diodes [13].

ESIPT occurs via an intramolecular hydrogen bonding way and it can be described by photophysical cycle with four energy levels. This model was proposed by Sengupta and Kasha in 1979 [14]. In this model, two levels correspond to the ground and the photoexcited states of the normal molecular form, and other two levels correspond to the ground and the excited states of the tautomer. The four energy level model is widely used in investigations of different aspects of ESIPT processes such as, for instance, effect of solvent on proton transfer dynamics [15] and impact of functional groups on ESIPT [16, 17]. An additional interesting issue is multiple ESIPT processes. There are works regarding double proton transfer in various organic compounds [18–22]. These studies consider molecules with two ESIPT sites. A review on photochemical features of compounds with possible excited state double proton transfer was performed by Serdiuk and Roshal in 2017 [23]. In some cases, the presence of double proton transfer causes triple emission from different excited state species [24, 25].

Another interesting issue concerns to multiple hydroxyl-containing compounds with one proton transfer site in the normal form. In this case, if several hydroxyl groups are located close to each other in a molecule, then the ESIPT process can lead to the next one. A proton donor site in the first ESIPT will be a proton acceptor during the second reaction. Therefore, two consecutive excited state proton transfers can occur. Such process can be called as a stepwise excited state intramolecular double proton transfer (stepwise ESIDPT) because it consists of two successive proton transfers. It can occur due to ultrafast dynamics of ESIPT, which take a short time in comparison with excited state lifetimes. Numerous experimental studies have demonstrated that single ESIPT reaction is characterized by ultrafast speed and it can take from less than 1 ps [26–29] to several tens of picoseconds [30]. For instance, the excited state proton transfer is completed in about 0.5–0.6 ps in the case of N-(3-pyridinyl)-2-pyridinecarboxamide [31], and it is even more faster in the case of 3-hydroxyflavone where a time constant of 35–60 fs can be assigned to this process depending on solvent [32]. At the same time, an excited state lifetime of the normal and tautomer form is higher than 100 ps. The measurements have shown that typically the excited state lifetime is in the range from several hundred picoseconds [28, 29, 33, 34] to several nanoseconds [35]. For instance, the excited state tautomer decay time constant is equal to about 850 ps in the case of 3-hydroxyflavone [28]. Therefore, it allows to assume that proton transfer reactions can occur successively in the excited state of compounds containing multiple adjacent hydroxyl groups due to a high rate of ESIPT.

**Fig. 1:**
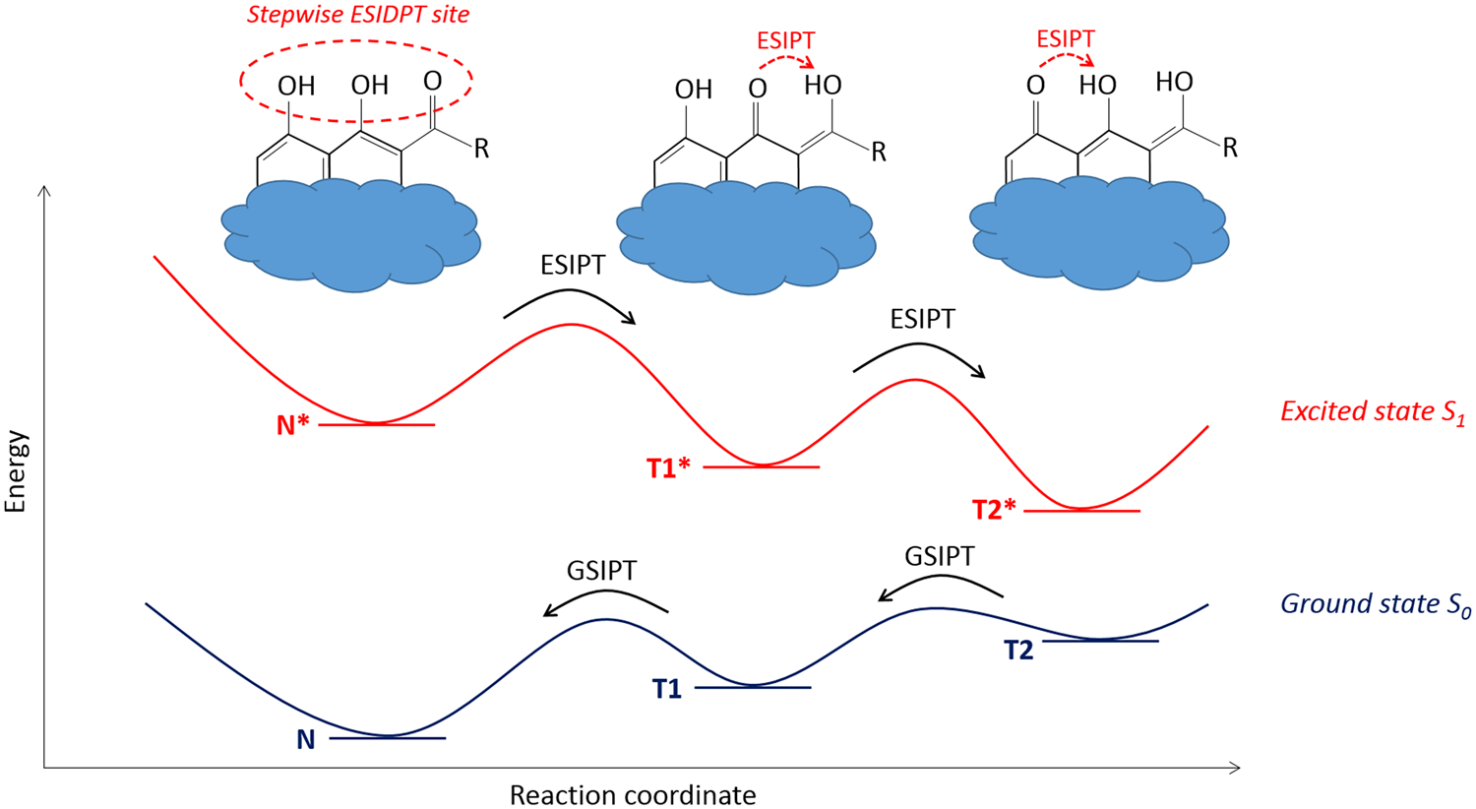
Schematic diagram of the stepwise ESIDPT consisted of two consecutive proton transfers. The ground states are denoted as N, T1 and T2 for the normal form, tautomer 1 and tautomer 2, respectively. The excited first-singlet states are denoted as N*, T1* and T2* for the normal form, tautomer 1 and tautomer 2, respectively. GSIPT means the ground state intramolecular proton transfer

Schematic diagram of the stepwise ESIDPT is depicted in Fig. 1. It presents two consecutive proton transfers within the molecule contained two adjacent hydroxyl groups. The stepwise double proton transfer occurs involving these hydroxyl groups and the adjacent carbonyl oxygen as a proton acceptor. Theoretically, the stepwise proton transfers can be consisted of more than two ESIPTs. It depends on molecular structure and number of involved hydroxyl groups. In this work, a formal analysis of fluorescence decay kinetics will be made in the case of a stepwise process consisting of two ESIPT reactions.

Experimental and theoretical study has shown that the stepwise ESIDPT occurs in 1,8-Dihydroxy-2-Naphthaldehyde [36, 37]. It is a naphthaldehyde derivative with two adjacent hydroxyl groups at positions 1 and 8. It was found that the first ESIPT is ultrafast and irreversible, and it takes less than 150 fs. The second ESIPT is reversible and the rates of forward and backward proton transfer are about (1.7 ps)^−1^ and (3.6 ps)^−1^, respectively [36]. Due to ultrafast character of first ESIPT, the rate of this process was faster than the time response of the detection system used in the work [36]. For this reason, the standard model with two species has been applied to analyse the fluorescence decays. This model considers the population kinetics of both tautomeric forms without considering the population of the normal form, because the excited state of the normal form has been almost depopulated in an ultrashort period after excitation [36]. This assumption is fully justified and based on the obtained experimental data for 1,8-Dihydroxy-2-Naphthaldehyde. However, the applied model cannot be expanded on stepwise ESIDPT processes in general, because it does not take into account kinetic rate of the first proton transfer. Therefore, in this work, a rigorous analysis of fluorescence decay kinetics is made using the model with three species, including a normal molecular form and two tautomers. The analysis proposed here can be applied in further researches focused on time-resolved spectroscopy of the stepwise ESIDPT systems.

## Theoretical Description

The formal theoretical analysis and the experimental investigation of excited state proton transfer was made by Loken et al. in 1972 [38] and then elaborated by Laws and Brand in the late 1970s [39]. The excited state decay kinetics of two species (naphthol and naphtholate) has been studied, and the theoretical model has been developed in their works [38–40]. Although the proposed model was not initially used to investigate the intramolecular process, but it can be modified and expanded to describe the stepwise ESIDPT processes. This model is widely applied to study ESIPT reactions both in theoretical and practical works [30, 33, 41–43]. For instance, the four levels model of ESIPT was used for theoretical investigation of proton transfer and studying substituent effects on ESIPT mechanism in different derivatives of 2-(2-Hydroxyphenyl)benzothiazole [17].

**Fig. 2:**
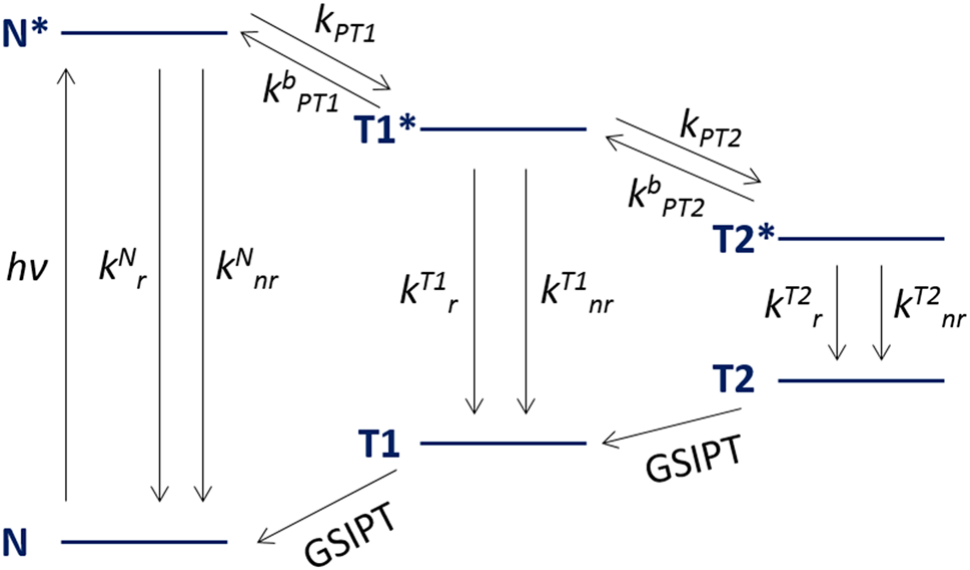
Six energy levels diagram of the stepwise ESIDPT process consisting of two consecutive proton transfers. The ground states are denoted as N, T1 and T2 for normal form, tautomer 1 and tautomer 2, respectively. The excited first-singlet states are denoted as N*, T1* and T2* for normal form, tautomer 1 and tautomer 2, respectively. ESIPT rate constants are denoted as *k*_*PT1*_ and *k*_*PT2*_. Backward ESIPT rate constants are denoted as *k*^*b*^_*PT1*_ and *k*^*b*^_*PT2*_. Rate constants of radiative and non-radiative relaxation are denoted by subscript *r* and *nr*, respectively. GSIPT means the ground state intramolecular proton transfer. Only the normal form N is directly excited by absorption of radiation *hν*

To discuss the stepwise process consisting of two ESIPT reactions, a three molecular species model will be used below. This model consists of six states: the three ground states and the three excited states. A six energy levels diagram with kinetic rate constants is presented in Fig. 2. In this model, the excited normal form N* is formed upon direct excitation (*hν* in the scheme) and can return to the ground state N by radiative relaxation (with the rate constant *k*^*N*^_*r*_) or non-radiative relaxation (*k*^*N*^_*nr*_). The excited normal form N* can also transform into the excited tautomer T1* due to forward ESIPT with the rate constant *k*_*PT1*_. The excited tautomer T1* can emit radiation (*k*^*T1*^_*r*_) or move to the ground state by non-radiative relaxation (*k*^*T1*^_*nr*_). T1* state can also return to N* state by reverse proton transfer. The rate constant of this backward ESIPT is denoted as *k*^*b*^_*PT1*_ in the scheme. However, if an adjacent hydroxyl group will be involved in a next proton transfer reaction, then T1* state can transform to the excited state of another tautomeric form T2* with the kinetic rate constant *k*_*PT2*_. The state T2* can fluoresce (*k*^*T2*^_*r*_) or move to the ground state by non-radiative relaxation (*k*^*T2*^_*nr*_). T2* state can also return to T1* state if the backward ESIPT (*k*^*b*^_*PT2*_) occurs.

In the following discussion, concentration of N*, T1* and T2* species will be denoted as [N*], [T1*] and [T2*], respectively. Then, the differential rate expressions for the change of concentration of these species are given by

1 $$\frac{d\left[{N}^{*}\right]}{dt}=-\left({k}_{r}^{N}+{k}_{nr}^{N}+{k}_{PT1}\right)\left[{N}^{*}\right]+{k}_{PT1}^{b}\left[{T1}^{*}\right]$$

2 $$\begin{array}{l}\frac{d\left[{T1}^{*}\right]}{dt}=-\left({k}_{r}^{T1}+{k}_{nr}^{T1}+{k}_{PT1}^{b}+{k}_{PT2}\right)\left[{T1}^{*}\right]+{k}_{PT1}\left[{N}^{*}\right]+{k}_{PT2}^{b}\left[{T2}^{*}\right]\end{array}$$

3 $$\frac{d\left[{T2}^{*}\right]}{dt}=-\left({k}_{r}^{T2}+{k}_{nr}^{T2}+{k}_{PT2}^{b}\right)\left[{T2}^{*}\right]+{k}_{PT2}\left[{T1}^{*}\right]$$

Considering all possible decay channels for each species, the following integral decay rate constants can be introduced:

4 $${K}_{N}={k}_{r}^{N}+{k}_{nr}^{N}+{k}_{PT1}$$

5 $${K}_{T1}={k}_{r}^{T1}+{k}_{nr}^{T1}+{k}_{PT1}^{b}+{k}_{PT2}$$

6 $${K}_{T2}={k}_{r}^{T2}+{k}_{nr}^{T2}+{k}_{PT2}^{b}$$

Then, the Eqs. (1)–(3) can be rewritten as

7 $$\frac{d\left[{N}^{*}\right]}{dt}=-{K}_{N}\left[{N}^{*}\right]+{k}_{PT1}^{b}\left[{T1}^{*}\right]$$

8 $$\frac{d\left[{T1}^{*}\right]}{dt}=-{K}_{T1}\left[{T1}^{*}\right]+{k}_{PT1}\left[{N}^{*}\right]+{k}_{PT2}^{b}\left[{T2}^{*}\right]$$

9 $$\frac{d\left[{T2}^{*}\right]}{dt}=-{K}_{T2}\left[{T2}^{*}\right]+{k}_{PT2}\left[{T1}^{*}\right]$$

The stepwise ESIDPT can be described by this system of the first order differential equations with the initial boundary condition that only the normal form is directly excited and populated at *t* = *0*. Therefore, the boundary condition can be written as follows

10 $${\left[{N}^{*}\right]}_{at\ t=0}=\left[{N}_{0}^{*}\right], {\left[{T1}^{*}\right]}_{at\ t=0}={\left[{T2}^{*}\right]}_{at\ t=0}=0$$

After making some mathematical operations, the characteristic equation of the system (7)–(9) can be found as

11 $${x}^{3}+{c}_{1} {x}^{2}+{c}_{2} x+{c}_{3}=0$$

where

12 $${c}_{1}={K}_{N}+{K}_{T1}+{K}_{T2}$$

13 $${c}_{2}={K}_{N}{ K}_{T1}+{K}_{N}{ K}_{T2}+{K}_{T1}{ K}_{T2}-{k}_{PT1} {k}_{PT1}^{b}-{k}_{PT2} {k}_{PT2}^{b}$$

14 $$\begin{aligned}{c}_{3}&={K}_{N}{ K}_{T1} { K}_{T2}-{{K}_{T2} k}_{PT1} {k}_{PT1}^{b}-{K}_{N} {k}_{PT2} {k}_{PT2}^{b}=\\& ={K}_{N} {k}_{PT2}\left({k}_{r}^{T2}+{k}_{nr}^{T2}\right)+{K}_{N} {K}_{T2}\left({k}_{r}^{T1}+{k}_{nr}^{T1}\right)\\&\quad+{K}_{T2} {k}_{PT1}^{b}\left({k}_{r}^{N}+{k}_{nr}^{N}\right)\end{aligned}$$

It is easily proven that ${c}_{1}$, ${c}_{2}$ and ${c}_{3}$ are positive (${c}_{1}, {c}_{2}, {c}_{3}>0$). Therefore, all real roots of the Eq. (11) are negative, what corresponds to decay of the excited state population for all species. As it was mentioned before, forward ESIPT reactions are ultrafast in comparison with excited state relaxation processes. It means that

15 $${k}_{PT1}, {k}_{PT2}\gg {k}_{r}^{N}, {k}_{nr}^{N}, {k}_{r}^{T1}, {k}_{nr}^{T1}, {k}_{r}^{T2}, {k}_{nr}^{T2}$$

The rate constants of the backward ESIPT (*k*^*b*^_*PT1*_ and *k*^*b*^_*PT2*_) can be either high (as the forward ESIPT rate) or low and even negligible [42]. It depends on compound properties and cannot be generalized. Then, taking into account the inequality (15), the expressions (12)–(14) can be rewritten as

16 $${c}_{1}\cong {k}_{PT1}+{k}_{PT1}^{b}+{k}_{PT2}+{k}_{PT2}^{b}$$

17 $${c}_{2}\cong {k}_{PT1} {k}_{PT2}+{k}_{PT1} {k}_{PT2}^{b}+{k}_{PT1}^{b} {k}_{PT2}^{b}$$

18 $$\begin{array}{l}{c}_{3}\cong {k}_{PT1} {k}_{PT2}\left({k}_{r}^{T2}+{k}_{nr}^{T2}\right)+{k}_{PT1} {k}_{PT2}^{b}\\\qquad\left({k}_{r}^{T1}+{k}_{nr}^{T1}\right)+{k}_{PT1}^{b} {k}_{PT2}^{b}\left({k}_{r}^{N}+{k}_{nr}^{N}\right)\end{array}$$

From algebra it is known that the characteristic cubic Eq. (11) has three distinct real roots if and only if its discriminant is positive [44]:

19 $$-4{c}_{1}^{3}{c}_{3}+{c}_{1}^{2}{c}_{2}^{2}+18\;{c}_{1}{c}_{2}{c}_{3}-4\;{c}_{2}^{3}-27\;{c}_{3}^{2}>0$$

Taking into account the inequality (15) and the expressions (16)–(18), it can be shown that ${c}_{1}^{2}{c}_{2}^{2}, {c}_{2}^{3}\gg {c}_{1}{c}_{2}{c}_{3}, {c}_{1}^{3}{c}_{3}$ and ${c}_{1}{c}_{2}{c}_{3}, {c}_{1}^{3}{c}_{3}\gg {c}_{3}^{2}$, and it can also be proven that ${c}_{1}^{2}{c}_{2}^{2}-4{c}_{2}^{3}>0$. Therefore, in the following we will consider a case where the condition (19) is satisfied. In this case, all three roots of the characteristic Eq. (11) are real negative numbers, which will be denoted further as ${x}_{1}$, ${x}_{2}$ and ${x}_{3}$ (${x}_{1}, {x}_{2}, {x}_{3}<0$).

Hence, the solution of the system (7)–(9) is given by

20 $$\left[{N}^{*}\right]\left(t\right)=\left[{N}_{0}^{*}\right]\left({\alpha }_{1}{e}^{-\frac{t}{{\tau }_{1}}}+{\alpha }_{2}{e}^{-\frac{t}{{\tau }_{2}}}+{\alpha }_{3}{e}^{-\frac{t}{{\tau }_{3}}}\right)$$

21 $$\left[{T1}^{*}\right]\left(t\right)=\left[{N}_{0}^{*}\right]\left({\beta }_{1}{e}^{-\frac{t}{{\tau }_{1}}}+{\beta }_{2}{e}^{-\frac{t}{{\tau }_{2}}}+{\beta }_{3}{e}^{-\frac{t}{{\tau }_{3}}}\right)$$

22 $$\left[{T2}^{*}\right]\left(t\right)=\left[{N}_{0}^{*}\right]\left({\gamma }_{1}{e}^{-\frac{t}{{\tau }_{1}}}+{\gamma }_{2}{e}^{-\frac{t}{{\tau }_{2}}}+{\gamma }_{3}{e}^{-\frac{t}{{\tau }_{3}}}\right)$$

where ${\tau }_{1}=-\frac{1}{{x}_{1}}$, ${\tau }_{2}=-\frac{1}{{x}_{2}}$ and ${\tau }_{3}=-\frac{1}{{x}_{3}}$; ${x}_{1}$, ${x}_{2}$ and ${x}_{3}$ (${x}_{1}, {x}_{2}, {x}_{3}<0$) are the roots of the characteristic Eq. (11). The pre-exponential factors are related by the following expressions:

23 $$\begin{array}{ccc}{\beta }_{i}=\frac{{K}_{N}-\frac{1}{{\tau }_{i}}}{{k}_{PT1}^{b}} {\alpha }_{i},& {\gamma }_{i}= \frac{{k}_{PT2}}{{K}_{T2}-\frac{1}{{\tau }_{i}}}{\beta }_{i}=\frac{{k}_{PT2}}{{k}_{PT1}^{b}} \frac{{K}_{N}-\frac{1}{{\tau }_{i}}}{{K}_{T2}-\frac{1}{{\tau }_{i}}} {\alpha }_{i}& (i=\overline{1, 3})\end{array}$$

To satisfy the initial boundary condition (10), the pre-exponential factors must also fulfil the following criteria:

24 $$\sum_{i=1}^{3}{\alpha }_{i}=1$$

25 $$\sum_{i=1}^{3}{\beta }_{i}=0 \Rightarrow \sum_{i=1}^{3}\frac{{\alpha }_{i}}{{\tau }_{i}}={K}_{N}$$

26 $$\sum_{i=1}^{3}{\gamma }_{i}=0 \Rightarrow \sum_{i=1}^{3}\frac{{K}_{N}-\frac{1}{{\tau }_{i}}}{{K}_{T2}-\frac{1}{{\tau }_{i}}}{\alpha }_{i}=0$$

Using Vieta's formulas [45] for cubic equations, the following expressions can be derived:

27 $$\frac{1}{{\tau }_{1}}+\frac{1}{{\tau }_{2}}+\frac{1}{{\tau }_{3}}={c}_{1}$$

28 $$\frac{1}{{\tau }_{1}{\tau }_{2}}+\frac{1}{{{\tau }_{1}\tau }_{3}}+\frac{1}{{{\tau }_{2}\tau }_{3}}={c}_{2}$$

29 $$\frac{1}{{{\tau }_{1}{\tau }_{2}\tau }_{3}}={c}_{3}$$

In turn, from (28) and (29), the expression for the sum of the three decay times can be found:

30 $${\tau }_{1}+{\tau }_{2}+{\tau }_{3}=\frac{{c}_{2}}{{c}_{3}}$$

Based on the Eqs. (20)–(22), the measured fluorescence intensity decays can be derived as

31 $$\begin{array}{l}{I}_{N}\left(t\right)={s}_{N} {k}_{r}^{N} \left[{N}^{*}\right]\left(t\right)={s}_{N} {k}_{r}^{N} \left[{N}_{0}^{*}\right]\\\qquad\qquad\left({\alpha }_{1}{e}^{-\frac{t}{{\tau }_{1}}}+{\alpha }_{2}{e}^{-\frac{t}{{\tau }_{2}}}+{\alpha }_{3}{e}^{-\frac{t}{{\tau }_{3}}}\right)\end{array}$$

32 $$\begin{array}{l}{I}_{T1}\left(t\right)={s}_{T1} {k}_{r}^{T1} \left[{T1}^{*}\right]\left(t\right)={s}_{T1} {k}_{r}^{T1} \left[{N}_{0}^{*}\right]\\\qquad\qquad\left({\beta }_{1}{e}^{-\frac{t}{{\tau }_{1}}}+{\beta }_{2}{e}^{-\frac{t}{{\tau }_{2}}}+{\beta }_{3}{e}^{-\frac{t}{{\tau }_{3}}}\right)\end{array}$$

33 $$\begin{array}{l}{I}_{T2}\left(t\right)={s}_{T2} {k}_{r}^{T2} \left[{T2}^{*}\right]\left(t\right)={s}_{T2} {k}_{r}^{T2} \left[{N}_{0}^{*}\right]\\\qquad\qquad\left({\gamma }_{1}{e}^{-\frac{t}{{\tau }_{1}}}+{\gamma }_{2}{e}^{-\frac{t}{{\tau }_{2}}}+{\gamma }_{3}{e}^{-\frac{t}{{\tau }_{3}}}\right)\end{array}$$

where ${I}_{N}$, ${I}_{T1}$ and ${I}_{T2}$ are the measured intensities of the N*, T1* and T2* form emission, respectively; ${s}_{N}$, ${s}_{T1}$ and ${s}_{T2}$ are the spectral sensitivities of the detection channels at wavelengths of the N*, T1* and T2* form emission, respectively.

Therefore, time-resolved fluorescence measurements and determining components of the emission decay kinetics allows to find parameters ${c}_{1}$, ${c}_{2}$ and ${c}_{3}$. In turn, they can be used to estimate the rate constants of relaxation and ESIPT processes from the Eqs. (12)–(14) or (16)–(18). If not the only normal form is directly excited and there are molecular tautomers before excitation, then the constraints (25) and (26) imposed by the boundary conditions should be changed.

It can be noted that, if the inequality (19) is violated, then the kinetics of the populations will be expressed by a single exponential decay with harmonic oscillations, and the characteristic time of exponential decay will be the same for all species.

## Examples of Compounds with Multiple Adjacent Hydroxyl Groups

In this section, two compounds containing multiple adjacent hydroxyl groups will be considered from the point of view of the possibility of ESIDPT process.

**Fig. 3:**
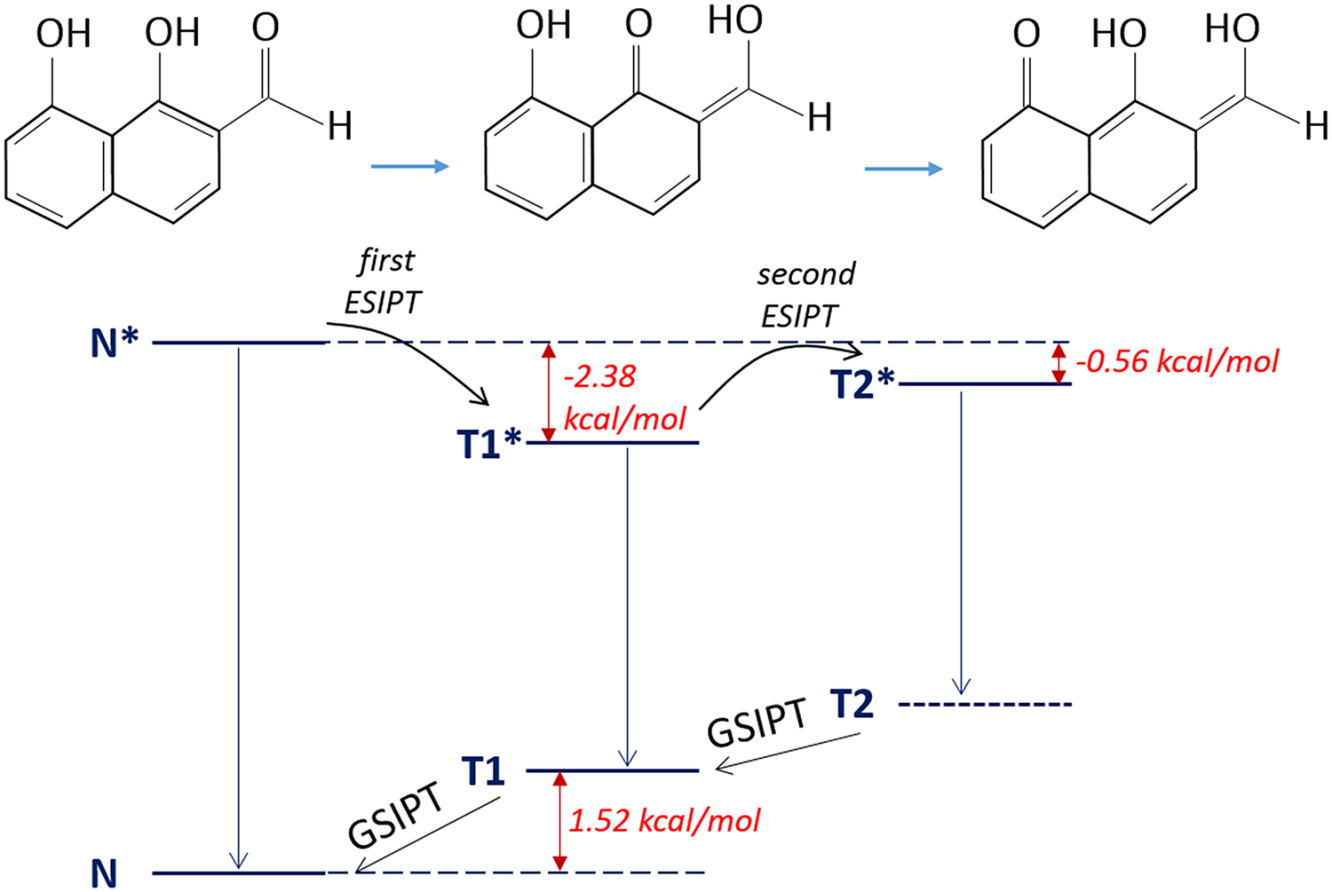
Energy diagram of the stepwise ESIDPT in 1,8-Dihydroxy-2-Naphthaldehyde in cyclohexane. Calculated relative energies are taken from the work of Peng et al. [36]. The calculations have been performed at the B3LYP/6–31 + G(d,p) and TD-B3LYP/6–31 + G(d,p) levels for the ground and excited states, respectively [36]

Theoretical and experimental studies have shown that ESIDPT occurs in 1,8-Dihydroxy-2-Naphthaldehyde [36, 37]. The schematic diagram of the proton transfer reactions is depicted in Fig. 3 It was found that the first ESIPT is ultrafast and irreversible, and it takes less than 150 fs. The second ESIPT is fast and reversible, and the rates of forward and backward proton transfer are about (1.7 ps)^−1^ and (3.6 ps)^−1^, respectively [36].

It is known, that the ESIPT reaction leads to strong changes in the molecular charge distribution [46, 47]. Therefore, in the case of stepwise ESIDPT, the charge redistribution should occur twice. For this reason, in some compounds with multiple adjacent hydroxyl groups just one ESIPT process occurs while a second proton transfer is blocked because of the impossibility of further charge redistribution in molecule.

The following example is tautomerization in scutellarein. The recent study has shown the possibility of ESIPT in scutellarein [48]. The structure of this flavone also contains three adjacent hydroxyl groups, that allows to consider a hypothetical ESIDPT process. As above mentioned, the first and second proton transfers are associated with charge redistribution within molecule. In the case of such benzopyran ring system as scutellarein, ESIDPT process causes a significant change in the electronic distribution. It will lead to a drastic growth of the dipole moment of a tautomeric form. It determines a high activation energy of tautomerization (from T1 to T2). At the same time, the calculations show that the difference between energies of T1 and T2 forms is equal to about 5 kcal/mol. Therefore, it could be assumed that second ESIPT can occur in scutellarein with low kinetic rate.

**Fig. 4:**
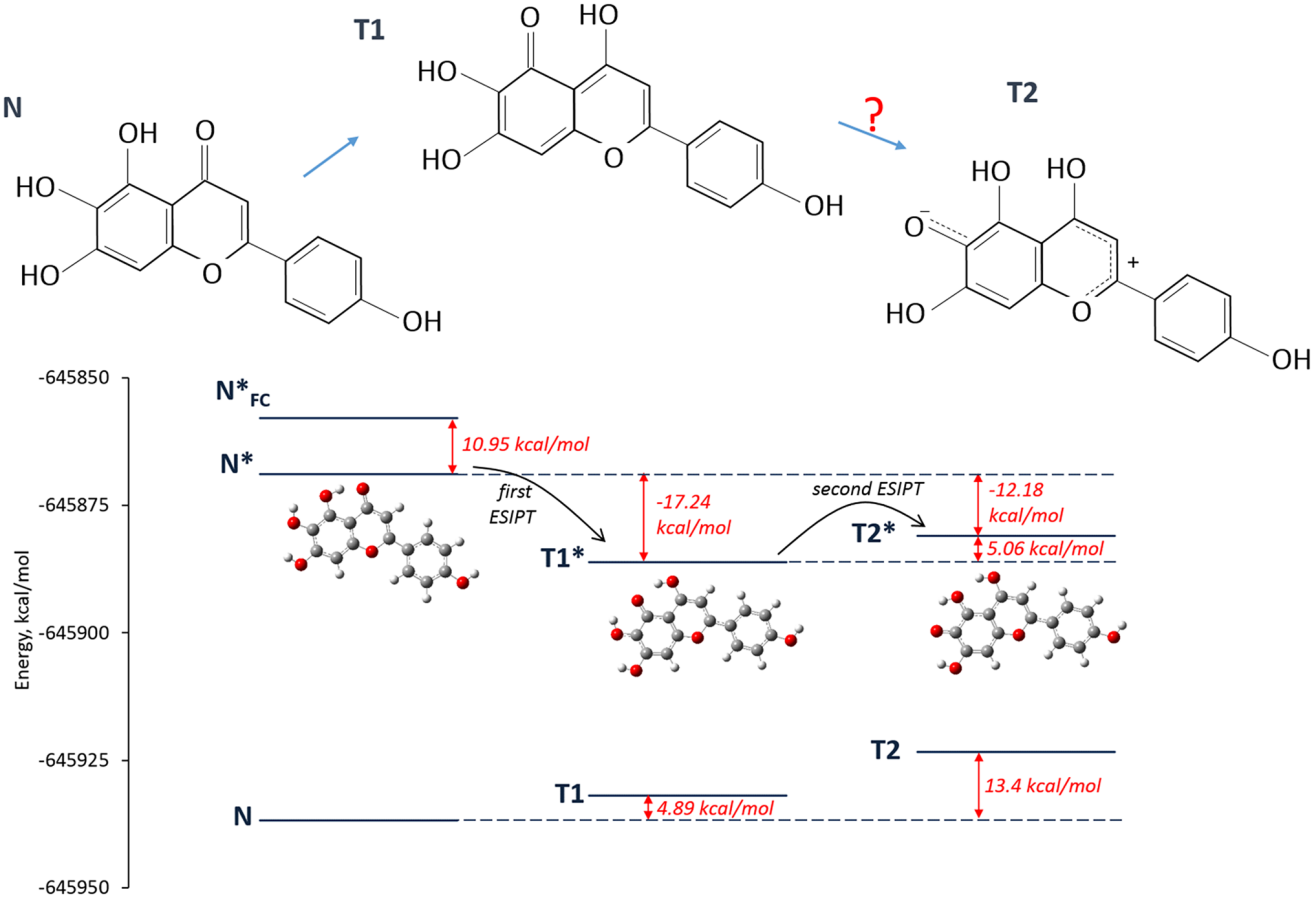
Energy diagram of the three different form of scutellarein in acetonitrile. N, T1 and T2 are denoted the ground state of normal form, tautomer 1 and tautomer 2, respectively. N*_FC_, N*, T1* and T2* are denoted the Franck–Condon state, excited relaxed normal form and excited tautomer 1 and tautomer 2, respectively. The detailed results of the quantum-chemical calculations for each form are given in the Supplementary Information

This prediction has been verified by quantum-chemical calculations of total energies for normal and tautomeric forms. The obtained results and hypothetical scheme of ESIDPT for scutellarein is presented in Fig. 4. The optimization of structures and energy calculations have been performed using Gaussian 09 program [49] based on Density Functional Theory (DFT) at the B3LYP/cc-pVTZ and TD-B3LYP/cc-pVTZ levels [50] for the ground and excited states, respectively. The Polarizable Continuum Model (PCM) together with the Integral Equation Formalism variant (IEF-PCM) [51] has been applied for modelling of solvent effects on molecular structure in acetonitrile.

The calculated total and relative energies and dipole moments are presented in Table 1. The detailed results of the quantum-chemical calculations and obtained optimized structures are given in the Supplementary Information.

**Table 1:** Calculated total and relative energies and dipole moments of different forms of scutellarein in acetonitrile

	Ground state			Excited state		
	Total energy, kcal/mol	Relative energy (to the N ground state), kcal/mol	Dipole moment, D	Total energy, kcal/mol	Relative energy (to the N ground state), kcal/mol	Dipole moment, D
Normal form, N	-645936.76	0	4.85	-645868.83	67.93	6.53
Normal form (the Franck–Condon state), N*_FC_	-	-	-	-645857.88	78.88	4.85
Tautomer 1, T1	-645931.87	4.89	8.84	-645886.07	50.69	11.26
Tautomer 2, T2	-645923.36	13.40	12.72	-645881.01	55.75	12.93

The calculations have been performed at the B3LYP/cc-pVTZ and TD-B3LYP/cc-pVTZ levels for the ground and excited states, respectively

However, it should be noted that, three-dimensional potential energy surfaces can provide additional information about transition states and about a way of proton-transfer process. Here, the quantum-chemical calculations have been limited to only studying the stable states (normal and tautomer forms) without transition states. This issue can be considered in detail in the further researches focused on this problem.

## Conclusions

The formal analysis of fluorescence decay kinetics has been made for compounds with stepwise excited state intramolecular double proton transfer (stepwise ESIDPT). Such processes can be observed in molecules with multiple adjacent hydroxyl groups. A three molecular species model has been used instead of model with two molecular forms in the case of single ESIPT. It considers normal form and two different tautomers. This model consists of six energy levels: the three ground states and the three excited states of molecular forms. The analytical solution shows that the fluorescence intensity kinetics are expressed by three-component exponential decays. It was shown that time-resolved fluorescence measurements will allow to estimate decay parameters that are directly related to kinetic rates of intramolecular proton transfers and relaxation processes of normal and tautomeric molecular forms. Moreover, the analytical relations between them and the emission decay parameters have been obtained in the work.

The obtained analytical results could be used in further experimental investigations of compounds with ESIDPT. Time-resolved emission spectroscopy together with the reported results could be used to estimate kinetic rates of proton transfers and, as a result, study of mechanism and reversibility of these intramolecular processes. However, due to ultrafast character of these processes, resolution of time-resolved measurements should be better than in the case of compounds with single ESIPT process.

The quantum-chemical calculations have been performed in the case of scutellarein. The calculated energies of excited states of normal form and tautomers have allowed to propose the hypothetical scheme of stepwise intramolecular proton transfers in this molecule.

## Supplementary Information

Below is the link to the electronic supplementary material.

Supplementary file1 (PDF 881 KB)

## Data Availability

The data that supports the findings of this study are available in the Supporting Information, and any additional data regarding the research are available from the corresponding author, D. Ushakou, upon a reasonable request.
