# Supplementary material for: Stepwise Excited-state Double Proton Transfer and Fluorescence Decay Analysis
Source: J Fluoresc. 2022 Oct 22;33(1):103–11. doi: 10.1007/s10895-022-03042-w (PMC9892138; doi:10.1007/s10895-022-03042-w)
Supplement: Supplementary file 1 — Supplementary file1 (PDF 881 KB) [file 10895_2022_3042_MOESM1_ESM.pdf]

Supplementary Information

for

“Stepwise excited-state double proton transfer and fluorescence decay analysis”

Tomasz Wróblewski, Dzmitryi Ushakou

Institute of Exact and Technical Sciences, Pomeranian University in Słupsk,
str. Arciszewskiego 22b, 76-200 Słupsk, Poland

Results of the quantum-chemical calculations for scutellarein in acetonitrile.

The optimization of structures and energy calculations have been performed using Gaussian 09 program[1] based on Density Functional Theory (DFT) at the B3LYP/cc-pVTZ and TD-B3LYP/cc-pVTZ (with 10 states) levels for the ground and excited states, respectively. The Polarizable Continuum Model (PCM) together with the Integral Equation Formalism variant (IEF-PCM) has been applied for modelling of solvent effects on molecular structure in acetonitrile.

**Ground state of the normal form (N)**	
Total energy	-645936.76 kcal/mol
Dipole moment	4.85 D
Optimized structure	
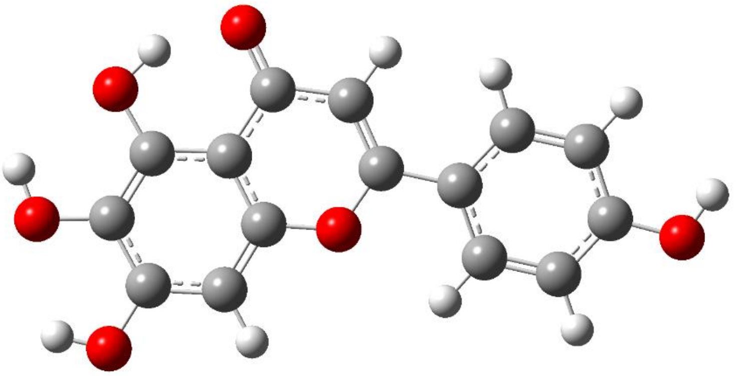 White, grey and red balls denote H, C and O atoms, respectively.	

[1] *Gaussian 09, Revision B.01*, M. J. Frisch, G. W. Trucks, H. B. Schlegel, G. E. Scuseria, M. A. Robb, J. R. Cheeseman, G. Scalmani, V. Barone, B. Mennucci, G. A. Petersson, H. Nakatsuji, M. Caricato, X. Li, H. P. Hratchian, A. F. Izmaylov, J. Bloino, G. Zheng, J. L. Sonnenberg, M. Hada, M. Ehara, K. Toyota, R. Fukuda, J. Hasegawa, M. Ishida, T. Nakajima, Y. Honda, O. Kitao, H. Nakai, T. Vreven, J. A. Montgomery, Jr., J. E. Peralta, F. Ogliaro, M. Bearpark, J. J. Heyd, E. Brothers, K. N. Kudin, V. N. Staroverov, T. Keith, R. Kobayashi, J. Normand, K. Raghavachari, A. Rendell, J. C. Burant, S. S. Iyengar, J. Tomasi, M. Cossi, N. Rega, J. M. Millam, M. Klene, J. E. Knox, J. B. Cross, V. Bakken, C. Adamo, J. Jaramillo, R. Gomperts, R. E. Stratmann, O. Yazyev, A. J. Austin, R. Cammi, C. Pomelli, J. W. Ochterski, R. L. Martin, K. Morokuma, V. G. Zakrzewski, G. A. Voth, P. Salvador, J. J. Dannenberg, S. Dapprich, A. D. Daniels, O. Farkas, J. B. Foresman, J. V. Ortiz, J. Cioslowski, D. J. Fox, *Gaussian, Inc., Wallingford CT*, 2010.

Distance matrix (angstroms)

		1	2	3	4	5
1	O	0.000000				
2	O	4.804755	0.000000			
3	O	5.503864	2.769168	0.000000		
4	O	4.717083	4.733478	2.703666	0.000000	
5	O	4.075352	2.588018	5.137050	6.350964	0.000000
6	O	6.258134	10.671713	11.761690	10.637722	8.865244
7	C	2.400972	2.406024	3.692243	4.142397	2.360121
8	C	1.367708	3.660014	4.137055	3.617343	3.615739
9	C	1.354298	5.050978	6.340728	5.948856	3.595397
10	C	2.819180	2.866644	4.906084	5.589025	1.256174
11	C	3.672890	1.349015	2.415380	3.643796	2.840194
12	C	2.345703	6.512467	7.729192	7.049561	4.884325
13	C	2.366723	4.291780	6.117400	6.319654	2.366706
14	C	2.350939	4.159596	3.654151	2.366231	4.794420
15	C	4.137463	2.350243	1.368577	2.383249	4.221958
16	C	3.617344	3.633336	2.367035	1.353927	5.019709
17	C	3.646705	7.273105	8.815817	8.359423	5.255198
18	C	2.712763	7.392289	8.188545	7.044472	6.078290
19	C	4.765150	8.648073	10.104643	9.451787	6.623435
20	C	4.092361	8.749039	9.560258	8.306969	7.296323
21	C	4.948967	9.315154	10.445444	9.434877	7.536998
22	H	3.341089	4.961991	7.040800	7.387052	2.626666
23	H	2.589729	5.238640	4.516314	2.609577	5.740606
24	H	4.037088	6.943898	8.775149	8.647580	4.695607
25	H	2.367893	7.150571	7.598337	6.200227	6.208441
26	H	5.732531	9.317539	10.951552	10.439417	7.123798
27	H	4.728338	9.492015	10.056353	8.547549	8.214987
28	H	4.680658	0.998141	3.745168	5.497239	1.675127
29	H	5.929196	2.286334	0.965948	3.660121	4.838957
30	H	5.487855	4.635053	2.174803	0.967305	6.580598
31	H	6.838741	11.046140	12.317901	11.347620	9.077484

		6	7	8	9	10
6	O	0.000000				
7	C	8.381445	0.000000			
8	C	7.624757	1.398773	0.000000		
9	C	5.626393	2.774200	2.369285	0.000000	
10	C	7.929475	1.446871	2.466493	2.441689	0.000000
11	C	9.778769	1.410292	2.413820	4.183303	2.491283
12	C	4.161714	4.230825	3.639386	1.464863	3.827386
13	C	6.534526	2.434671	2.765673	1.361328	1.433652
14	C	8.390136	2.437427	1.384704	3.607295	3.751314
15	C	10.394561	2.420364	2.769995	4.987221	3.752296
16	C	9.764933	2.788470	2.381495	4.749773	4.235122
17	C	3.663554	5.180795	4.845925	2.497291	4.432669
18	C	3.623247	5.006681	4.070202	2.494077	4.927075
19	C	2.415470	6.512625	6.052440	3.768907	5.812693
20	C	2.354453	6.374647	5.449012	3.767153	6.199600
21	C	1.356650	7.030075	6.310574	4.270757	6.579301
22	H	6.375276	3.413375	3.843169	2.120499	2.159622
23	H	8.064491	3.417088	2.152738	3.942938	4.618592
24	H	4.530765	5.089371	5.047230	2.727199	4.089096
25	H	4.479995	4.752676	3.606143	2.707795	4.984556
26	H	2.677471	7.304152	6.973212	4.636016	6.455414
27	H	2.566065	7.093089	6.034067	4.639494	7.072616
28	H	10.200054	2.356841	3.745372	4.655843	2.271353
29	H	12.146790	3.841868	4.574388	6.599128	4.848821
30	H	11.527232	4.547762	4.282557	6.649841	5.976519

31	H	0.964727	8.830461	8.192179	6.056605	8.237219

		11	12	13	14	15
11	C	0.000000				
12	C	5.636273	0.000000			
13	C	3.749979	2.520438	0.000000		
14	C	2.810889	4.685780	4.148265	0.000000	
15	C	1.384059	6.364116	4.853144	2.432025	0.000000
16	C	2.411071	5.960319	5.004541	1.390865	1.401323
17	C	6.582152	1.401535	3.003113	5.994986	7.470851
18	C	6.350041	1.403070	3.766268	4.776222	6.828752
19	C	7.920493	2.425341	4.382362	7.094415	8.747103
20	C	7.728077	2.426881	4.938989	6.095533	8.203477
21	C	8.430277	2.805913	5.193390	7.143365	9.077015
22	H	4.634740	2.781562	1.077696	5.225240	5.826408
23	H	3.890020	4.739781	4.761762	1.079155	3.409988
24	H	6.437477	2.160133	2.711279	6.318022	7.475713
25	H	5.991639	2.152847	4.058819	4.043429	6.269138
26	H	8.697332	3.403513	5.033898	8.076297	9.608828
27	H	8.393329	3.408933	5.883692	6.477628	8.725734
28	H	1.876632	6.081279	3.690467	4.540313	3.148501
29	H	2.441528	8.036244	6.176045	4.341983	1.913642
30	H	3.758293	7.833349	6.868843	3.166838	2.380978
31	H	10.239231	4.609276	6.812450	9.055391	10.949724

		16	17	18	19	20
16	C	0.000000				
17	C	7.213472	0.000000			
18	C	6.149694	2.403923	0.000000		
19	C	8.379765	1.382475	2.776563	0.000000	
20	C	7.480171	2.777778	1.382401	2.413364	0.000000
21	C	8.502648	2.406196	2.404671	1.396284	1.394935
22	H	6.063387	2.730533	4.157630	4.056028	5.130465
23	H	2.150227	6.115693	4.512444	7.062821	5.725508
24	H	7.418010	1.079908	3.392314	2.119907	3.857389
25	H	5.429692	3.386502	1.079102	3.855623	2.127111
26	H	9.328401	2.140027	3.858863	1.082315	3.395086
27	H	7.864116	3.858592	2.146887	3.388908	1.080876
28	H	4.287691	6.685952	7.100561	8.067582	8.415804
29	H	3.173195	9.020281	8.635539	10.351884	10.017202
30	H	1.901331	9.105640	7.919378	10.249053	9.207161
31	H	10.410863	3.830289	4.319114	2.458712	3.169924

		21	22	23	24	25
21	C	0.000000				
22	H	5.095044	0.000000			
23	H	6.902497	5.814901	0.000000		
24	H	3.374425	2.096166	6.605491	0.000000	
25	H	3.377305	4.674590	3.619367	4.294193	0.000000
26	H	2.152321	4.506302	8.096809	2.436305	4.937922
27	H	2.141933	6.154704	5.942226	4.938107	2.459886
28	H	8.848750	4.207187	5.602647	6.246501	7.002029
29	H	10.808738	7.008076	5.287925	8.865526	8.138154
30	H	10.300954	7.912744	3.550054	9.316327	7.108928
31	H	1.917440	6.504487	8.807520	4.524716	5.259823

		26	27	28	29	30
26	H	0.000000				
27	H	4.282758	0.000000			
28	H	8.659153	9.236746	0.000000		

```
   29  H   11.137144  10.589549   3.281777   0.000000
   30  H   11.211928   9.482235   5.506741   3.130844   0.000000
   31  H    2.303778   3.512192  10.502353  12.644641  12.214099

                31
   31  H    0.000000
```

Ground state of the tautomer 1 (T1)

Total energy	-645931.87 kcal/mol
Dipole moment	8.84 D

Optimized structure

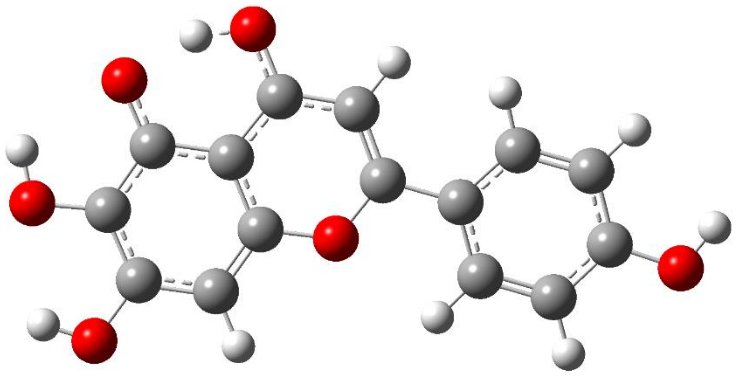

White, grey and red balls denote H, C and O atoms, respectively.

Distance matrix (angstroms)

		1	2	3	4	5
1	O	0.000000				
2	O	4.776433	0.000000			
3	O	5.518480	2.770352	0.000000		
4	O	4.728752	4.736510	2.730035	0.000000	
5	O	4.072996	2.496069	5.068396	6.309533	0.000000
6	O	6.254155	10.616233	11.771321	10.663577	8.865067
7	C	2.398000	2.379098	3.679404	4.125728	2.349002
8	C	1.371041	3.652452	4.147456	3.605103	3.622745
9	C	1.347598	5.007734	6.338647	5.950239	3.605160
10	C	2.760646	2.820595	4.859061	5.529399	1.312350
11	C	3.711509	1.292853	2.402312	3.670546	2.778234
12	C	2.342858	6.462450	7.730663	7.060744	4.888337
13	C	2.365788	4.227434	6.092113	6.309198	2.369529
14	C	2.361921	4.158499	3.680691	2.367245	4.780345
15	C	4.147993	2.358362	1.371246	2.378463	4.178690
16	C	3.632901	3.621280	2.376017	1.356086	4.975953
17	C	3.646298	7.208458	8.806922	8.370514	5.253851
18	C	2.710481	7.357241	8.207279	7.065819	6.086799
19	C	4.764261	8.583805	10.100486	9.468863	6.621885
20	C	4.089860	8.709156	9.579821	8.333208	7.302351
21	C	4.947146	9.262030	10.454346	9.458853	7.538982
22	H	3.343170	4.879493	7.000918	7.372650	2.622015
23	H	2.621378	5.236976	4.538943	2.602431	5.738572
24	H	4.038659	6.870063	8.755528	8.653940	4.690644
25	H	2.364631	7.129551	7.628059	6.223837	6.219640
26	H	5.732333	9.246347	10.941336	10.455548	7.120039
27	H	4.726801	9.461059	10.086600	8.580571	8.223320
28	H	4.452174	1.512915	4.214556	5.795017	1.040497
29	H	5.887012	2.237437	0.969831	3.680585	4.703922
30	H	5.501004	4.649414	2.205929	0.967221	6.537360
31	H	6.837587	10.986557	12.323615	11.373618	9.078228

		6	7	8	9	10
6	O	0.000000				
7	C	8.372006	0.000000			
8	C	7.624536	1.409015	0.000000		
9	C	5.617447	2.771916	2.376570	0.000000	
10	C	7.889013	1.404348	2.425000	2.401939	0.000000
11	C	9.797441	1.431374	2.467066	4.203266	2.460072

12	C	4.157768	4.224552	3.643723	1.459879	3.786495
13	C	6.530390	2.426869	2.777556	1.370871	1.408433
14	C	8.402292	2.433317	1.373083	3.611410	3.697008
15	C	10.400222	2.412906	2.776985	4.987589	3.709883
16	C	9.787075	2.769707	2.376569	4.753036	4.173577
17	C	3.660805	5.171555	4.853326	2.494029	4.400318
18	C	3.619633	5.004763	4.071075	2.489803	4.883831
19	C	2.414180	6.503160	6.058069	3.763819	5.779445
20	C	2.352379	6.370519	5.448969	3.760909	6.156565
21	C	1.354272	7.022853	6.313126	4.264244	6.540428
22	H	6.382328	3.400543	3.854153	2.134837	2.152664
23	H	8.106057	3.419879	2.149445	3.967587	4.573430
24	H	4.527613	5.079359	5.058283	2.727408	4.066654
25	H	4.476847	4.754166	3.603383	2.704412	4.939269
26	H	2.676983	7.293645	6.980277	4.631825	6.427589
27	H	2.563832	7.091637	6.033098	4.633967	7.028843
28	H	9.723798	2.281789	3.686088	4.270818	1.870715
29	H	12.088311	3.773575	4.536951	6.534297	4.748718
30	H	11.551004	4.535545	4.276603	6.652385	5.924943
31	H	0.964907	8.822363	8.195842	6.050710	8.202942

		11	12	13	14	15
11	C	0.000000				
12	C	5.654621	0.000000			
13	C	3.741749	2.522033	0.000000		
14	C	2.865661	4.694684	4.149485	0.000000	
15	C	1.405130	6.366064	4.838912	2.445189	0.000000
16	C	2.436172	5.971705	4.993099	1.406806	1.388932
17	C	6.586227	1.402573	2.996738	6.006005	7.467257
18	C	6.384942	1.404348	3.773460	4.787372	6.841048
19	C	7.927515	2.424835	4.376363	7.107083	8.746184
20	C	7.760110	2.426088	4.942458	6.107963	8.215682
21	C	8.450106	2.804380	5.191653	7.156735	9.083282
22	H	4.608687	2.794069	1.076686	5.225881	5.802796
23	H	3.944123	4.774992	4.782602	1.078693	3.417169
24	H	6.429330	2.162129	2.699862	6.329103	7.466641
25	H	6.038713	2.153947	4.069717	4.053003	6.287762
26	H	8.696990	3.403325	5.025499	8.089497	9.604816
27	H	8.434181	3.408599	5.889537	6.491612	8.744261
28	H	2.129285	5.651615	3.194428	4.623549	3.510886
29	H	2.356464	7.972364	6.083363	4.330606	1.886333
30	H	3.780070	7.843836	6.858137	3.172487	2.379192
31	H	10.253699	4.608055	6.808865	9.070013	10.954456

		16	17	18	19	20
16	C	0.000000				
17	C	7.222174	0.000000			
18	C	6.172521	2.406732	0.000000		
19	C	8.393530	1.381471	2.778168	0.000000	
20	C	7.505754	2.779192	1.380853	2.415498	0.000000
21	C	8.523967	2.405625	2.404141	1.396909	1.395983
22	H	6.046866	2.731527	4.176818	4.058228	5.146623
23	H	2.163747	6.155507	4.548731	7.105796	5.764488
24	H	7.420966	1.079694	3.395470	2.118071	3.858607
25	H	5.457175	3.388953	1.078962	3.857099	2.126052
26	H	9.340097	2.139212	3.860316	1.082160	3.396896
27	H	7.896460	3.859845	2.145699	3.390497	1.080733
28	H	4.523321	6.164369	6.746049	7.542994	8.026658
29	H	3.154571	8.940507	8.593917	10.276212	9.974613
30	H	1.900994	9.114480	7.940668	10.263469	9.232613

31	H	10.432510	3.829953	4.318058	2.459524	3.169789

		21	22	23	24	25
21	C	0.000000				
22	H	5.104446	0.000000			
23	H	6.945138	5.838512	0.000000		
24	H	3.373392	2.083039	6.644870	0.000000	
25	H	3.377178	4.696654	3.649974	4.297233	0.000000
26	H	2.152755	4.503333	8.141158	2.434285	4.939243
27	H	2.142512	6.173444	5.980468	4.939152	2.459385
28	H	8.381805	3.615348	5.661106	5.672487	6.736074
29	H	10.749960	6.896428	5.278366	8.772296	8.113583
30	H	10.323011	7.895717	3.544984	9.319800	7.133723
31	H	1.917084	6.510668	8.852498	4.523852	5.258841

		26	27	28	29	30
26	H	0.000000				
27	H	4.283883	0.000000			
28	H	8.095079	8.890023	0.000000		
29	H	11.052668	10.561135	3.749286	0.000000	
30	H	11.224701	9.515014	5.888695	3.172362	0.000000
31	H	2.305250	3.510825	9.990657	12.579468	12.237494

		31
31	H	0.000000

Ground state of the tautomer 2 (T2)

Total energy	-645923.36 kcal/mol
Dipole moment	12.72 D

Optimized structure

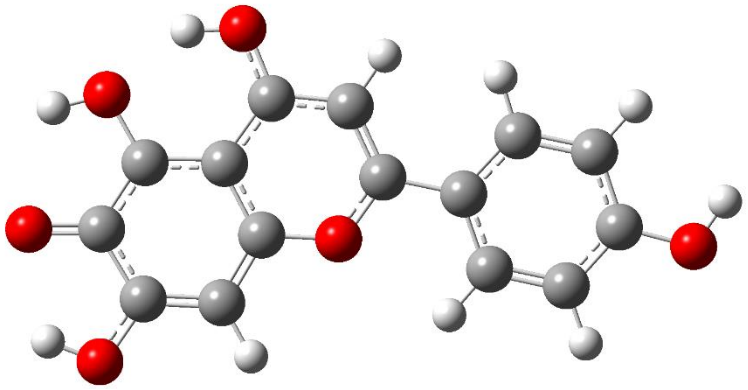

White, grey and red balls denote H, C and O atoms, respectively.

Distance matrix (angstroms)

		1	2	3	4	5
1	O	0.000000				
2	O	4.813072	0.000000			
3	O	5.466637	2.742606	0.000000		
4	O	4.733086	4.707859	2.650208	0.000000	
5	O	4.074195	2.666032	5.173160	6.389671	0.000000
6	O	6.260505	10.685728	11.726958	10.664146	8.826432
7	C	2.410785	2.404118	3.642025	4.127326	2.400078
8	C	1.360533	3.682630	4.106439	3.620795	3.654018
9	C	1.338588	5.078018	6.309848	5.954982	3.598295
10	C	2.745891	2.902175	4.860576	5.539100	1.329813
11	C	3.669686	1.374123	2.361246	3.596564	2.926850
12	C	2.342775	6.530104	7.693727	7.062701	4.863688
13	C	2.348977	4.294320	6.067858	6.305120	2.334157
14	C	2.348667	4.188661	3.640084	2.385805	4.846408
15	C	4.183822	2.367977	1.283878	2.340306	4.321664
16	C	3.605131	3.657126	2.362405	1.343460	5.079555
17	C	3.645785	7.283918	8.780141	8.375999	5.206112
18	C	2.717367	7.417521	8.157726	7.064090	6.076258
19	C	4.766504	8.658909	10.069464	9.473417	6.572580
20	C	4.096970	8.770743	9.528567	8.330975	7.282383
21	C	4.952711	9.331394	10.412312	9.460295	7.502610
22	H	3.328658	4.946336	6.980911	7.367933	2.546235
23	H	2.583466	5.268532	4.516355	2.667524	5.781116
24	H	4.036263	6.950740	8.740858	8.663331	4.628339
25	H	2.374502	7.181455	7.570324	6.218696	6.226257
26	H	5.733767	9.324563	10.916066	10.461869	7.059752
27	H	4.735667	9.517702	10.028218	8.575670	8.210461
28	H	4.529814	1.809664	4.474924	6.027517	0.977635
29	H	5.536017	0.974072	2.180163	4.546509	3.624552
30	H	5.434738	4.473179	2.008100	0.981969	6.498911
31	H	6.843497	11.060541	12.285443	11.376584	9.031676

		6	7	8	9	10
6	O	0.000000				
7	C	8.398226	0.000000			
8	C	7.620534	1.419525	0.000000		
9	C	5.614512	2.800245	2.368502	0.000000	
10	C	7.888034	1.413805	2.414527	2.405081	0.000000
11	C	9.785674	1.403690	2.419361	4.201257	2.500834

12	C	4.158488	4.249736	3.636211	1.456235	3.785188
13	C	6.541118	2.433811	2.756594	1.377622	1.394832
14	C	8.380238	2.454214	1.384798	3.601099	3.706409
15	C	10.443736	2.456377	2.823359	5.042086	3.776409
16	C	9.749625	2.785659	2.372210	4.739252	4.196268
17	C	3.661485	5.195016	4.845854	2.491702	4.396621
18	C	3.619360	5.031988	4.067452	2.487328	4.885839
19	C	2.414635	6.527515	6.051791	3.761196	5.776387
20	C	2.352417	6.396983	5.445354	3.757504	6.157103
21	C	1.354365	7.048965	6.308647	4.261263	6.539299
22	H	6.399772	3.402151	3.833309	2.139662	2.133782
23	H	8.040389	3.432951	2.148175	3.921884	4.562586
24	H	4.527980	5.100649	5.050859	2.727084	4.061404
25	H	4.476286	4.781676	3.602360	2.703314	4.944061
26	H	2.677798	7.316589	6.973624	4.629431	6.423158
27	H	2.563440	7.118188	6.031043	4.630734	7.030498
28	H	9.697391	2.423263	3.824157	4.299657	1.896496
29	H	11.551581	3.175422	4.301940	5.937312	3.850504
30	H	11.507013	4.420675	4.208068	6.576349	5.821162
31	H	0.964897	8.848634	8.191842	6.048650	8.201738

		11	12	13	14	15
11	C	0.000000				
12	C	5.646299	0.000000			
13	C	3.752265	2.530159	0.000000		
14	C	2.814580	4.677538	4.139160	0.000000	
15	C	1.396020	6.414363	4.887963	2.485532	0.000000
16	C	2.402221	5.946315	4.986485	1.385867	1.436088
17	C	6.592538	1.403074	3.005204	5.993676	7.521714
18	C	6.361308	1.405285	3.782954	4.762889	6.880214
19	C	7.930101	2.425377	4.385849	7.091730	8.798297
20	C	7.737112	2.426356	4.952160	6.082567	8.253551
21	C	8.439636	2.805043	5.201907	7.136075	9.128435
22	H	4.627584	2.805520	1.076751	5.215656	5.850871
23	H	3.894458	4.717344	4.744897	1.079878	3.464745
24	H	6.450040	2.163038	2.706646	6.323433	7.527294
25	H	6.003137	2.155089	4.078650	4.023624	6.319265
26	H	8.707029	3.403786	5.034176	8.076517	9.659967
27	H	8.403055	3.408897	5.899296	6.463004	8.775848
28	H	2.436814	5.652701	3.158404	4.824816	3.801089
29	H	1.882747	7.393544	5.235005	4.538722	2.295340
30	H	3.586446	7.777480	6.752591	3.138420	2.198312
31	H	10.249690	4.609447	6.820185	9.050928	11.002462

		16	17	18	19	20
16	C	0.000000				
17	C	7.205123	0.000000			
18	C	6.132573	2.407620	0.000000		
19	C	8.370420	1.381712	2.778479	0.000000	
20	C	7.462908	2.779486	1.380202	2.415540	0.000000
21	C	8.489770	2.405894	2.404323	1.396668	1.396468
22	H	6.041853	2.742715	4.191906	4.072146	5.163082
23	H	2.155823	6.102863	4.480408	7.048027	5.695367
24	H	7.415773	1.079707	3.396771	2.117953	3.858926
25	H	5.408489	3.390129	1.079103	3.857547	2.125394
26	H	9.321613	2.139211	3.860668	1.082197	3.397088
27	H	7.846087	3.860151	2.144966	3.390460	1.080747
28	H	4.784899	6.112284	6.781886	7.488068	8.044586
29	H	3.720685	8.207629	8.202793	9.576555	9.571145
30	H	1.838736	9.043658	7.892346	10.200652	9.190739

31	H	10.400575	3.831329	4.318386	2.460681	3.170118

		21	22	23	24	25
21	C	0.000000				
22	H	5.120831	0.000000			
23	H	6.880940	5.799582	0.000000		
24	H	3.373340	2.087715	6.601375	0.000000	
25	H	3.377368	4.711277	3.575520	4.299057	0.000000
26	H	2.152585	4.515208	8.086106	2.433634	4.939732
27	H	2.142833	6.190682	5.909078	4.939478	2.458274
28	H	8.360208	3.504295	5.842923	5.583441	6.810131
29	H	10.198563	5.915966	5.598930	7.907604	7.881345
30	H	10.273392	7.785474	3.591041	9.240755	7.094553
31	H	1.917333	6.528028	8.789670	4.524866	5.258881

		26	27	28	29	30
26	H	0.000000				
27	H	4.284003	0.000000			
28	H	8.012114	8.926242	0.000000		
29	H	10.267543	10.268508	2.727070	0.000000	
30	H	11.158351	9.485021	5.998572	4.117290	0.000000
31	H	2.306880	3.510542	9.942598	11.958469	12.188770

		31
31	H	0.000000

**Excited state of the normal form (N^*_{FC}, the Franck-Condon state)**	
Total energy	-645857.88 kcal/mol
Dipole moment	4.85 D

Structure (the Franck-Condon state)

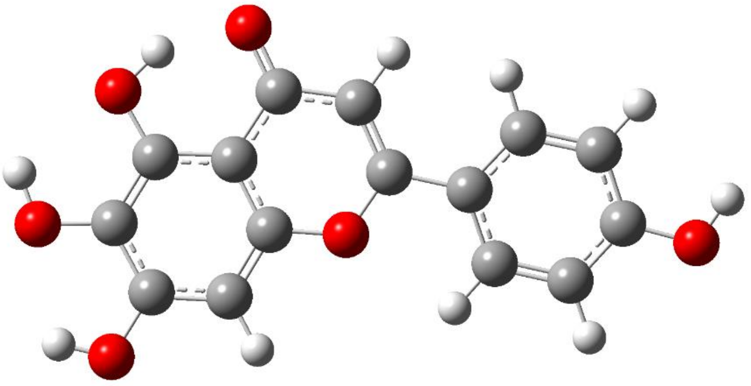

White, grey and red balls denote H, C and O atoms, respectively.

**Excited state of the normal form (N*, the relaxed excited state)**	
Total energy	-645868.83 kcal/mol
Dipole moment	6.53 D

Optimized structure

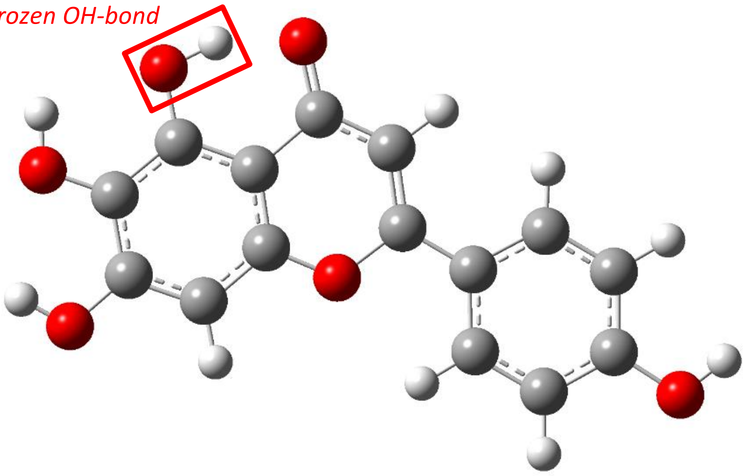

White, grey and red balls denote H, C and O atoms, respectively.
The indicated OH bond length was frozen during optimization.

Distance matrix (angstroms)

		1	2	3	4	5
1	O	0.000000				
2	O	4.719562	0.000000			
3	O	5.449521	2.741766	0.000000		
4	O	4.716501	4.753756	2.749057	0.000000	
5	O	4.137519	2.465103	5.040683	6.379254	0.000000
6	O	6.303955	10.619860	11.752796	10.675398	8.979331
7	C	2.366527	2.357089	3.682737	4.203136	2.367588
8	C	1.339571	3.582372	4.112224	3.669565	3.628270
9	C	1.426909	4.991998	6.337049	6.023675	3.668292
10	C	2.851250	2.813583	4.896594	5.671894	1.291165
11	C	3.621212	1.307949	2.409657	3.701135	2.778927
12	C	2.368653	6.413018	7.680306	7.073986	4.938865
13	C	2.436031	4.194372	6.073500	6.379424	2.404974

14	C	2.352805	4.091114	3.605554	2.363815	4.814551
15	C	4.122360	2.353494	1.327389	2.401180	4.214619
16	C	3.620890	3.632678	2.358885	1.351315	5.052429
17	C	3.708311	7.212910	8.803463	8.423141	5.347507
18	C	2.719719	7.310077	8.151604	7.055468	6.152231
19	C	4.810311	8.583289	10.083742	9.497496	6.718324
20	C	4.101851	8.667415	9.524343	8.314946	7.375364
21	C	4.982856	9.248216	10.422433	9.464339	7.634103
22	H	3.409108	4.883778	7.003944	7.450855	2.691562
23	H	2.568915	5.170543	4.486685	2.604428	5.759112
24	H	4.119351	6.904360	8.782194	8.739448	4.798609
25	H	2.353348	7.072709	7.562594	6.197098	6.279379
26	H	5.784560	9.259756	10.936290	10.495225	7.221896
27	H	4.720498	9.404722	10.011862	8.532410	8.289553
28	H	4.585653	0.998141	3.718178	5.514980	1.523731
29	H	5.876769	2.269892	0.971106	3.712340	4.718820
30	H	5.501054	4.704010	2.272366	0.966277	6.625150
31	H	6.881561	10.986731	12.301834	11.385942	9.182524

		6	7	8	9	10
6	O	0.000000				
7	C	8.374449	0.000000			
8	C	7.640649	1.373402	0.000000		
9	C	5.630861	2.768228	2.395048	0.000000	
10	C	7.949484	1.468761	2.485540	2.451042	0.000000
11	C	9.762523	1.401010	2.377492	4.168582	2.486943
12	C	4.208134	4.179930	3.618058	1.422808	3.800695
13	C	6.600051	2.406949	2.776189	1.398276	1.391114
14	C	8.444340	2.446984	1.425080	3.679364	3.803369
15	C	10.425423	2.469439	2.784835	5.035035	3.812761
16	C	9.810667	2.851940	2.433978	4.827233	4.320675
17	C	3.683310	5.169413	4.861591	2.483103	4.429987
18	C	3.645584	4.970207	4.055746	2.485172	4.929680
19	C	2.431730	6.493143	6.057082	3.748940	5.808440
20	C	2.371972	6.338762	5.436231	3.752733	6.199235
21	C	1.371688	7.008478	6.312378	4.259981	6.584352
22	H	6.442773	3.399446	3.855350	2.135938	2.129353
23	H	8.099724	3.410097	2.170076	3.994973	4.654685
24	H	4.545159	5.099465	5.082462	2.724899	4.093097
25	H	4.496043	4.719496	3.589995	2.716181	4.999754
26	H	2.687404	7.290399	6.983072	4.616179	6.449636
27	H	2.580164	7.050824	6.012193	4.626065	7.072578
28	H	10.129380	2.289879	3.652998	4.569406	2.182848
29	H	12.133754	3.830794	4.546499	6.586656	4.826801
30	H	11.574965	4.636808	4.348155	6.739898	6.083721
31	H	0.963585	8.815595	8.201071	6.047553	8.243185

		11	12	13	14	15
11	C	0.000000				
12	C	5.576717	0.000000			
13	C	3.701474	2.540110	0.000000		
14	C	2.783931	4.714067	4.200667	0.000000	
15	C	1.435737	6.362096	4.875854	2.400324	0.000000
16	C	2.456921	5.985761	5.065575	1.377490	1.408117
17	C	6.561138	1.420089	3.040483	6.059510	7.511240
18	C	6.304823	1.420515	3.814500	4.799395	6.827312
19	C	7.891909	2.445010	4.419922	7.145393	8.775388
20	C	7.683395	2.447448	4.984769	6.119325	8.202058
21	C	8.399543	2.837173	5.244246	7.185817	9.095544
22	H	4.599591	2.802855	1.079569	5.279385	5.858755

23	H	3.863100	4.755319	4.804454	1.079599	3.384368
24	H	6.437187	2.173641	2.744390	6.403345	7.541576
25	H	5.949375	2.165715	4.114353	4.054746	6.260497
26	H	8.674626	3.421327	5.065622	8.134363	9.645824
27	H	8.341708	3.426866	5.929809	6.484190	8.709257
28	H	1.833436	5.959342	3.562481	4.472466	3.158495
29	H	2.438031	7.979700	6.116760	4.300917	1.901378
30	H	3.854899	7.869649	6.944121	3.170247	2.429203
31	H	10.215261	4.642861	6.862129	9.106500	10.977239

		16	17	18	19	20
16	C	0.000000				
17	C	7.280034	0.000000			
18	C	6.165160	2.414410	0.000000		
19	C	8.429952	1.380135	2.777871	0.000000	
20	C	7.493899	2.779004	1.382767	2.406928	0.000000
21	C	8.538495	2.413564	2.411719	1.398341	1.393655
22	H	6.129372	2.759927	4.209148	4.092047	5.180137
23	H	2.133053	6.164313	4.516637	7.096778	5.731218
24	H	7.511851	1.080190	3.405840	2.116691	3.858813
25	H	5.431803	3.400121	1.078606	3.856339	2.125008
26	H	9.388144	2.134647	3.861395	1.083524	3.390025
27	H	7.857094	3.860969	2.142956	3.384815	1.082010
28	H	4.290349	6.601490	7.000079	7.981489	8.315668
29	H	3.181631	8.998141	8.596283	10.323238	9.978612
30	H	1.914506	9.184158	7.940493	10.308631	9.223968
31	H	10.455836	3.836493	4.331276	2.465862	3.177406

		21	22	23	24	25
21	C	0.000000				
22	H	5.148143	0.000000			
23	H	6.926646	5.857840	0.000000		
24	H	3.379228	2.107666	6.674874	0.000000	
25	H	3.380095	4.734158	3.607134	4.313772	0.000000
26	H	2.152771	4.532751	8.138420	2.425966	4.939853
27	H	2.140073	6.207680	5.928833	4.940662	2.449380
28	H	8.762311	4.102580	5.527895	6.180834	6.908848
29	H	10.780979	6.955604	5.262760	8.859982	8.103634
30	H	10.341404	7.994426	3.546839	9.425852	7.115113
31	H	1.922318	6.554194	8.841099	4.524404	5.267636

		26	27	28	29	30
26	H	0.000000				
27	H	4.280253	0.000000			
28	H	8.580004	9.133291	0.000000		
29	H	11.112625	10.545037	3.264710	0.000000	
30	H	11.282910	9.473933	5.572977	3.231182	0.000000
31	H	2.306709	3.521514	10.423449	12.622544	12.263621

		31
31	H	0.000000

Excited state of the tautomer 1 (T1*)

Total energy	-645886.07 kcal/mol
Dipole moment	11.26 D

Optimized structure

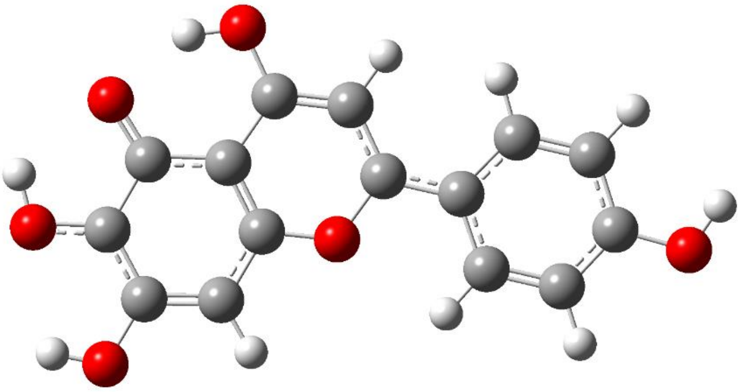

White, grey and red balls denote H, C and O atoms, respectively.

Distance matrix (angstroms)

		1	2	3	4	5
1	O	0.000000				
2	O	4.757899	0.000000			
3	O	5.445469	2.670136	0.000000		
4	O	4.707447	4.727354	2.768770	0.000000	
5	O	4.140933	2.691084	5.177668	6.495660	0.000000
6	O	6.301666	10.692593	11.746997	10.641457	8.919255
7	C	2.382253	2.384264	3.677264	4.216889	2.426704
8	C	1.354063	3.585986	4.095114	3.668011	3.657200
9	C	1.402533	5.069382	6.338456	6.004817	3.656950
10	C	2.791168	2.933901	4.916960	5.665339	1.350015
11	C	3.672480	1.260813	2.385661	3.734266	2.904366
12	C	2.367015	6.495908	7.687558	7.058292	4.909786
13	C	2.411086	4.293539	6.087622	6.378044	2.373071
14	C	2.341633	4.087348	3.607101	2.366200	4.880295
15	C	4.114399	2.324910	1.331103	2.402521	4.348420
16	C	3.612920	3.613119	2.367621	1.354452	5.160278
17	C	3.697278	7.300800	8.806955	8.404267	5.283445
18	C	2.727012	7.375129	8.150384	7.029094	6.133478
19	C	4.805338	8.670540	10.087145	9.476462	6.651320
20	C	4.109171	8.735225	9.522305	8.285241	7.345613
21	C	4.983155	9.325070	10.421440	9.436610	7.581452
22	H	3.388288	4.984583	7.015328	7.448621	2.609431
23	H	2.538022	5.166388	4.497323	2.615582	5.802294
24	H	4.102530	6.999055	8.787800	8.725006	4.715955
25	H	2.365562	7.121759	7.555938	6.165345	6.278393
26	H	5.777140	9.351880	10.940737	10.475424	7.141146
27	H	4.732901	9.465688	10.008373	8.499865	8.268775
28	H	4.516305	1.808869	4.420261	6.066440	0.980621
29	H	5.807497	2.113876	0.977372	3.727171	4.784722
30	H	5.489028	4.659544	2.288328	0.966122	6.750124
31	H	6.879777	11.068654	12.300880	11.356860	9.115137

		6	7	8	9	10
6	O	0.000000				
7	C	8.394437	0.000000			
8	C	7.652710	1.368695	0.000000		
9	C	5.624927	2.790785	2.392044	0.000000	
10	C	7.909276	1.450590	2.428106	2.415802	0.000000
11	C	9.817344	1.436321	2.409099	4.226060	2.534161

12	C	4.197237	4.209618	3.632686	1.427849	3.769175
13	C	6.587575	2.420925	2.761166	1.399370	1.368439
14	C	8.422290	2.455556	1.424325	3.653536	3.764746
15	C	10.415896	2.471082	2.764232	5.033942	3.827932
16	C	9.789731	2.862538	2.426788	4.813452	4.311495
17	C	3.678165	5.187581	4.864341	2.485144	4.398564
18	C	3.639680	4.999309	4.077261	2.481062	4.890670
19	C	2.427620	6.513959	6.065688	3.750988	5.777132
20	C	2.367201	6.367394	5.457933	3.750329	6.161507
21	C	1.367695	7.032205	6.327018	4.258143	6.547711
22	H	6.438041	3.404676	3.839934	2.144614	2.115187
23	H	8.047467	3.412768	2.167533	3.940424	4.595618
24	H	4.540399	5.111083	5.076965	2.728530	4.067645
25	H	4.492119	4.747702	3.614907	2.706754	4.957111
26	H	2.685101	7.308113	6.988257	4.618976	6.421746
27	H	2.576038	7.081671	6.038884	4.622996	7.034417
28	H	9.746562	2.389205	3.747522	4.305147	1.889471
29	H	12.064988	3.746592	4.461800	6.521375	4.781128
30	H	11.542358	4.641957	4.337927	6.721993	6.082716
31	H	0.963891	8.837011	8.213282	6.046236	8.208926

		11	12	13	14	15
11	C	0.000000				
12	C	5.641239	0.000000			
13	C	3.754722	2.538442	0.000000		
14	C	2.829043	4.698633	4.182827	0.000000	
15	C	1.448819	6.366377	4.889286	2.390367	0.000000
16	C	2.493546	5.978113	5.065161	1.375594	1.403398
17	C	6.614995	1.415944	3.032850	6.038091	7.512387
18	C	6.367321	1.416672	3.808073	4.783140	6.821976
19	C	7.948157	2.440637	4.412685	7.126334	8.776035
20	C	7.745692	2.442954	4.978531	6.103334	8.195867
21	C	8.458129	2.830302	5.235628	7.167096	9.090991
22	H	4.641356	2.807726	1.078822	5.261047	5.870253
23	H	3.907562	4.707939	4.762729	1.079169	3.378523
24	H	6.484585	2.171530	2.736577	6.380952	7.545653
25	H	6.009285	2.162173	4.106099	4.038153	6.248907
26	H	8.727746	3.417176	5.058366	8.114550	9.647835
27	H	8.405313	3.422945	5.924081	6.471358	8.701145
28	H	2.340898	5.647098	3.159001	4.769478	3.770056
29	H	2.320649	7.921117	6.059608	4.254186	1.863846
30	H	3.868821	7.855217	6.944007	3.167293	2.425269
31	H	10.271874	4.635595	6.853272	9.086629	10.972878

		16	17	18	19	20
16	C	0.000000				
17	C	7.268156	0.000000			
18	C	6.150102	2.411816	0.000000		
19	C	8.417878	1.380467	2.777280	0.000000	
20	C	7.477460	2.778429	1.382583	2.408088	0.000000
21	C	8.522460	2.411703	2.409907	1.397801	1.393729
22	H	6.127561	2.760306	4.209611	4.091944	5.181419
23	H	2.136687	6.112280	4.468501	7.046575	5.684252
24	H	7.502084	1.080196	3.403252	2.116267	3.858244
25	H	5.412434	3.396502	1.078733	3.855914	2.125999
26	H	9.376659	2.135486	3.860446	1.083166	3.390869
27	H	7.839982	3.860086	2.143784	3.385361	1.081706
28	H	4.784315	6.156054	6.777133	7.533493	8.050201
29	H	3.158788	8.933101	8.534479	10.259129	9.916640
30	H	1.911282	9.166879	7.914970	10.289265	9.195315

31	H	10.439011	3.835196	4.328280	2.464559	3.175384

		21	22	23	24	25
21	C	0.000000				
22	H	5.147087	0.000000			
23	H	6.876901	5.815925	0.000000		
24	H	3.377350	2.106537	6.623546	0.000000	
25	H	3.379629	4.732196	3.559335	4.309607	0.000000
26	H	2.152637	4.532069	8.087721	2.426314	4.939074
27	H	2.140151	6.209050	5.887150	4.939781	2.452654
28	H	8.393628	3.551428	5.779044	5.652570	6.790961
29	H	10.716117	6.894548	5.229651	8.794987	8.041083
30	H	10.315035	7.993407	3.555308	9.413213	7.083810
31	H	1.921208	6.552861	8.790409	4.523445	5.266019

		26	27	28	29	30
26	H	0.000000				
27	H	4.280444	0.000000			
28	H	8.071433	8.920811	0.000000		
29	H	11.048225	10.485470	3.922701	0.000000	
30	H	11.265065	9.442125	6.194973	3.261914	0.000000
31	H	2.306712	3.518723	9.990661	12.556693	12.236202

		31
31	H	0.000000

Excited state of the tautomer 2 (T2*)	
Total energy	-645881.01 kcal/mol
Dipole moment	12.93 D

Optimized structure

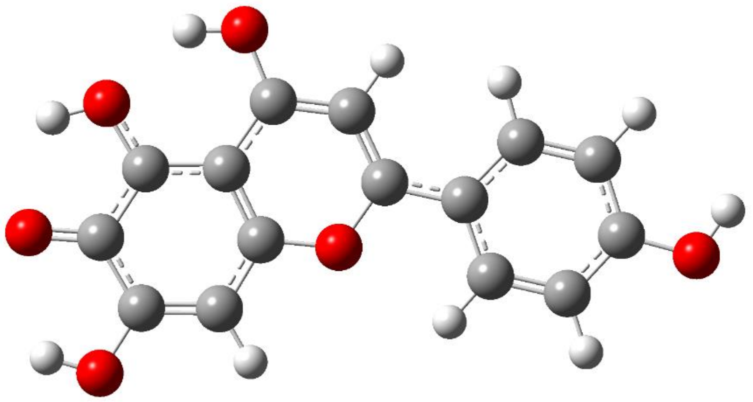

White, grey and red balls denote H, C and O atoms, respectively.

Distance matrix (angstroms)

		1	2	3	4	5
1	O	0.000000				
2	O	4.791623	0.000000			
3	O	5.450328	2.644796	0.000000		
4	O	4.740625	4.729973	2.763987	0.000000	
5	O	4.155178	2.745158	5.197074	6.534955	0.000000
6	O	6.279352	10.691235	11.728858	10.673218	8.864565
7	C	2.407091	2.392211	3.648483	4.220177	2.453614
8	C	1.377260	3.608083	4.075244	3.667483	3.691187
9	C	1.393619	5.073210	6.311592	6.016429	3.633471
10	C	2.802491	2.923528	4.868480	5.652051	1.353585
11	C	3.662253	1.330727	2.336975	3.674299	2.992553
12	C	2.361733	6.508337	7.676089	7.090135	4.879249
13	C	2.397418	4.301063	6.054842	6.374932	2.356650
14	C	2.355855	4.107618	3.606096	2.384965	4.910336
15	C	4.184403	2.347676	1.266188	2.383196	4.429247
16	C	3.626343	3.638867	2.380922	1.354097	5.205177
17	C	3.689184	7.301259	8.783607	8.428197	5.231748
18	C	2.710377	7.386535	8.142777	7.065515	6.105492
19	C	4.794577	8.671000	10.066633	9.505686	6.596716
20	C	4.092542	8.746113	9.515707	8.324060	7.312094
21	C	4.966495	9.328859	10.406550	9.470213	7.533972
22	H	3.373959	4.980763	6.975553	7.443423	2.561641
23	H	2.540129	5.188021	4.506742	2.651652	5.825062
24	H	4.097347	6.992902	8.756289	8.741110	4.654170
25	H	2.343507	7.133912	7.551829	6.203418	6.258623
26	H	5.768087	9.349934	10.917279	10.502825	7.081116
27	H	4.716232	9.480151	10.008635	8.544633	8.240395
28	H	4.605772	1.929699	4.520964	6.199313	0.967442
29	H	5.482343	4.582021	2.219196	0.970997	6.719569
30	H	6.862259	11.068332	12.282513	11.389252	9.058748
31	H	5.511782	0.982225	2.052686	4.550958	3.708493

		6	7	8	9	10
6	O	0.000000				
7	C	8.390444	0.000000			
8	C	7.653662	1.385159	0.000000		
9	C	5.620779	2.788895	2.397604	0.000000	
10	C	7.905957	1.433489	2.432873	2.417114	0.000000
11	C	9.790494	1.420670	2.375262	4.205892	2.531666

12	C	4.183752	4.218572	3.649061	1.437302	3.777899
13	C	6.563247	2.416385	2.762007	1.378739	1.385875
14	C	8.423718	2.460751	1.409947	3.649623	3.753362
15	C	10.463333	2.515285	2.810136	5.078445	3.851902
16	C	9.786605	2.866277	2.413673	4.806705	4.297954
17	C	3.671095	5.188228	4.876474	2.491638	4.404223
18	C	3.632707	5.004235	4.083758	2.479831	4.891093
19	C	2.423045	6.514842	6.076789	3.756264	5.781792
20	C	2.361611	6.372443	5.464243	3.751478	6.163832
21	C	1.362476	7.033234	6.333567	4.259275	6.549470
22	H	6.412191	3.394165	3.840422	2.128640	2.123776
23	H	8.046970	3.417597	2.151284	3.933017	4.584133
24	H	4.534610	5.108004	5.088975	2.734948	4.073523
25	H	4.487511	4.750424	3.614141	2.698558	4.951429
26	H	2.681721	7.308662	7.000634	4.625829	6.428496
27	H	2.571935	7.088518	6.043996	4.623449	7.036300
28	H	9.729517	2.493904	3.863567	4.331708	1.917346
29	H	11.545292	4.579243	4.289980	6.683691	6.000356
30	H	0.964281	8.835840	8.218992	6.046968	8.210490
31	H	11.556859	3.177901	4.231247	5.938314	3.882934

		11	12	13	14	15
11	C	0.000000				
12	C	5.630066	0.000000			
13	C	3.755193	2.522730	0.000000		
14	C	2.778092	4.709734	4.169222	0.000000	
15	C	1.437504	6.424730	4.928710	2.433629	0.000000
16	C	2.445853	5.985377	5.054039	1.371193	1.433227
17	C	6.604524	1.412457	3.020004	6.043347	7.562136
18	C	6.342231	1.412980	3.784258	4.791459	6.877214
19	C	7.935187	2.435356	4.398661	7.133077	8.827284
20	C	7.721409	2.438429	4.957595	6.111609	8.251167
21	C	8.437083	2.822018	5.215931	7.172850	9.142979
22	H	4.643717	2.787899	1.078420	5.247271	5.903633
23	H	3.858091	4.715138	4.743271	1.080658	3.420624
24	H	6.479437	2.169624	2.730402	6.383198	7.589645
25	H	5.975011	2.158740	4.077295	4.044388	6.301361
26	H	8.719245	3.412922	5.049409	8.121113	9.697979
27	H	8.378237	3.419316	5.902468	6.481545	8.757893
28	H	2.531139	5.663091	3.170262	4.894376	3.935443
29	H	3.743006	7.844063	6.881543	3.153124	2.316642
30	H	10.252086	4.625952	6.835410	9.090876	11.022867
31	H	1.857452	7.375322	5.249273	4.446389	2.255270

		16	17	18	19	20
16	C	0.000000				
17	C	7.269264	0.000000			
18	C	6.153967	2.410989	0.000000		
19	C	8.420070	1.379489	2.778000	0.000000	
20	C	7.481307	2.779544	1.382716	2.411515	0.000000
21	C	8.523688	2.409891	2.407681	1.399149	1.393649
22	H	6.114837	2.741783	4.185001	4.073761	5.159466
23	H	2.135574	6.115012	4.474043	7.051243	5.689734
24	H	7.500412	1.080178	3.401857	2.115346	3.859382
25	H	5.414339	3.394236	1.078569	3.856514	2.127218
26	H	9.378738	2.135934	3.860884	1.082885	3.393445
27	H	7.845901	3.860904	2.145225	3.388218	1.081421
28	H	4.928058	6.130920	6.808097	7.504763	8.070533
29	H	1.877018	9.142523	7.921174	10.275392	9.208375
30	H	10.438690	3.831790	4.324599	2.462501	3.173085

31	H	3.680036	8.229664	8.171723	9.591896	9.545419

		21	22	23	24	25
21	C	0.000000				
22	H	5.125286	0.000000			
23	H	6.880315	5.797360	0.000000		
24	H	3.376356	2.093167	6.623690	0.000000	
25	H	3.378717	4.704239	3.563490	4.306014	0.000000
26	H	2.153940	4.519536	8.092193	2.427558	4.939391
27	H	2.140273	6.186791	5.894845	4.940620	2.456882
28	H	8.385502	3.508837	5.897601	5.601303	6.841977
29	H	10.315793	7.925623	3.578117	9.373529	7.098987
30	H	1.919756	6.533227	8.792504	4.521441	5.263946
31	H	10.196559	5.957926	5.511097	7.956384	7.831714

		26	27	28	29	30
26	H	0.000000				
27	H	4.282251	0.000000			
28	H	8.025150	8.953224	0.000000		
29	H	11.246025	9.470261	6.251952	0.000000	
30	H	2.305138	3.516192	9.962694	12.235274	0.000000
31	H	10.297305	10.227360	2.844815	4.207058	11.967427

		31
31	H	0.000000
